# Title Oxidative Stress in Age-Related Macular Degeneration: From Molecular Mechanisms to Emerging Therapeutic Targets

**DOI:** 10.3390/antiox14101251

**Published:** 2025-10-18

**Authors:** Tatsuya Mimura, Hidetaka Noma

**Affiliations:** 1 Department of Ophthalmology, Tsurumi University School of Dental Medicine, Yokohama 230-0063, Japan; 2 Department of Ophthalmology, Tokyo Medical University Ibaraki Medical Center, Ami-cho 300-0395, Japan

**Keywords:** age-related macular degeneration, oxidative stress, reactive oxygen species (ROS), mitochondrial dysfunction, antioxidant therapy

## Abstract

Age-related macular degeneration (AMD) is a leading cause of irreversible visual impairment in the elderly, and oxidative stress, primarily mediated by reactive oxygen species (ROS), is widely recognized as a central driver of its onset and progression. The retina is highly susceptible to oxidative damage due to its elevated oxygen consumption, abundant polyunsaturated fatty acids, and continuous exposure to light. Recent studies have elucidated molecular mechanisms in which mitochondrial dysfunction, disruption of redox homeostasis, inflammation, and complement activation interact to promote degeneration of retinal pigment epithelium (RPE) and photoreceptor cells. In addition to age-related oxidative stress, environmental factors such as motor vehicle exhaust and volatile organic compounds (VOCs) can accelerate the accumulation of lipofuscin and drusen, thereby fostering a chronic pro-inflammatory milieu. From a therapeutic perspective, beyond conventional antioxidant supplementation, emerging strategies targeting oxidative stress-related pathways have gained attention, including mitochondrial protectants, activation of the nuclear factor erythroid 2-related factor 2 (Nrf2) pathway, anti-inflammatory agents, and gene therapy. Importantly, several innovative approaches are under investigation, such as saffron supplementation with neuroprotective properties, drug repositioning of levodopa, and nanotechnology-based delivery systems to enhance retinal bioavailability of antioxidants and gene therapies. This review summarizes the pathophysiological role of oxidative stress in AMD from a molecular mechanistic perspective and discusses recent advances in research and novel therapeutic targets.

## 1. Introduction

Age-related macular degeneration (AMD) is one of the leading causes of irreversible central vision loss in aging populations and ranks among the most prevalent causes of visual impairment worldwide. Its burden is projected to increase further with global population aging. In the United States, approximately 10% of individuals aged ≥ 65 years and 25% of those aged ≥ 75 years are affected by AMD, highlighting its high prevalence among older adults [1]. In an Australian cohort of participants aged ≥ 49 years (*n* = 3654), the 5-year incidence was reported as 22.7% for early AMD and 6.8% for late AMD [2].

**Figure 1:**
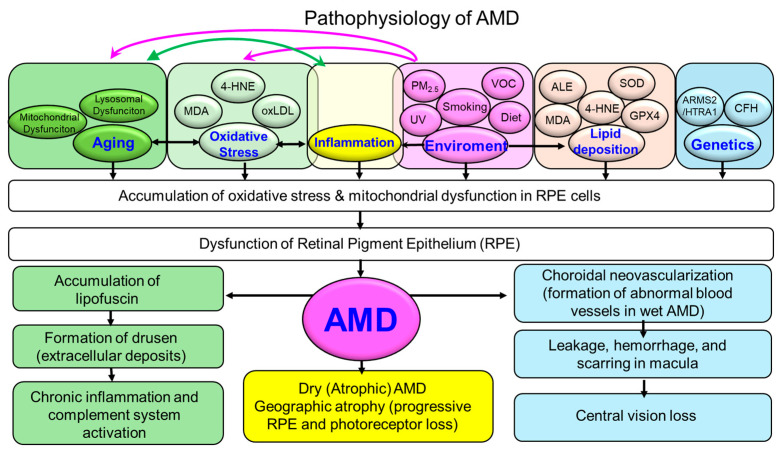
Pathophysiological mechanisms of AMD. Schematic illustration summarizing the pathophysiological cascade of AMD. Schematic illustration of the pathophysiological cascade in AMD. Aging, genetic predisposition, and environmental risk factors (e.g., smoking, diet, light exposure, volatile organic compounds: VOCs) converge to induce mitochondrial dysfunction, oxidative stress, and impaired antioxidant defenses in RPE cells. These processes promote lipofuscin accumulation, disrupted photoreceptor support, and drusen formation (extracellular deposits). Oxidative stress also interacts bidirectionally with chronic inflammation, lipid peroxidation, and complement activation, amplifying cellular damage. Disease progression diverges into two major phenotypes: dry (atrophic) AMD, characterized by RPE and photoreceptor loss with geographic atrophy, and wet (neovascular) AMD, marked by choroidal neovascularization, vascular leakage, hemorrhage, and macular scarring. Both pathways ultimately culminate in progressive central vision loss. 4-HNE = 4-hydroxy-2-nonenal; ALE = Advanced lipoxidation end product; AMD = Age-related macular degeneration; ARMS2 = Age-related maculopathy susceptibility 2; CFH = Complement factor H; GPX4 = Glutathione peroxidase 4; HTRA1 = HtrA serine peptidase 1; MDA = Malondialdehyde; oxLDL = Oxidized low-density lipoprotein; PM2.5 = Particulate matter with an aerodynamic diameter of less than 2.5 μm; RPE = Retinal pigment epithelium; SOD = Superoxide dismutase; UV = Ultraviolet; VOC = Volatile organic compound.

As illustrated in Figure 1, the etiology of AMD is highly multifactorial. In addition to aging—the most prominent risk factor—genetic, environmental, and lifestyle factors interact in complex ways to drive disease onset and progression. Variants in complement-related genes, such as complement factor H (CFH) and age-related maculopathy susceptibility 2/HtrA serine peptidase 1 (ARMS2/HTRA1), have been shown to increase susceptibility, influencing diverse pathological mechanisms including lipid metabolism, angiogenesis, and maintenance of the retinal extracellular matrix [1].

Among these factors, oxidative stress has emerged as a central contributor to AMD pathogenesis. Retinal pigment epithelium (RPE) cells and photoreceptors are particularly vulnerable to oxidative damage due to their high metabolic activity, intense exposure to light, and enrichment in oxygen and polyunsaturated fatty acids (PUFAs) [3]. Accumulation of reactive oxygen species (ROS) has been shown to impair RPE function, promote lipofuscin and drusen formation, and trigger chronic inflammation and complement activation—hallmark features of AMD [4,5]. The pathogenic role of oxidative stress is further supported by retinal degeneration observed in Sod1- or Sod2-deficient mouse models [6].

Although the epidemiology and fundamental pathophysiology of AMD have been extensively described in previous reviews, several critical knowledge gaps remain unresolved. First, the precise contribution of gene–environment interactions in shaping oxidative stress susceptibility is poorly understood. While genetic variants are firmly established as risk factors, their interaction with modifiable exposures, including smoking and dietary antioxidants, requires further clarification [7]. Second, the cross-talk between oxidative stress and chronic inflammation in AMD progression remains incompletely defined. Experimental data suggest bidirectional amplification loops between ROS and complement activation, yet their temporal sequence in human disease is unclear [8]. Third, despite numerous proposed oxidative biomarkers, lack of standardized validation protocols hampers their translation into clinical practice [9]. Finally, while emerging experimental platforms, such as patient-derived induced pluripotent stem cell (iPSC)-RPE cells and retinal organoids, have the potential to model oxidative stress responses and screen novel therapeutics, they remain underutilized in AMD research [10]. Addressing these gaps is essential for moving beyond descriptive pathology toward mechanism-driven, personalized interventions for AMD.

This review provides an overview of the pathophysiology of AMD and the molecular basis of oxidative stress, with a focus on their interrelationship from the perspectives of biomarkers and therapeutic targets. We also summarize recent findings from animal models and clinical studies and discuss both the potential and challenges of therapeutic interventions targeting oxidative stress. Future research directions include standardization of oxidative stress-related biomarkers, integration into personalized medicine approaches, and long-term evaluation of treatment efficacy. A deeper understanding of oxidative stress in AMD will be pivotal for the development of novel diagnostic and therapeutic strategies.

## 2. Retinal Vulnerability to Oxidative Stress

The macula is a specialized region of the retina responsible for high-acuity vision, color discrimination, and fine spatial resolution. Its unique anatomical and metabolic characteristics underlie its pronounced susceptibility to oxidative stress. The fovea, in particular, is densely packed with cone photoreceptors and exhibits exceptionally high oxygen consumption and metabolic demand. Moreover, the foveal avascular zone has limited retinal vasculature and relies predominantly on the choroidal circulation for nutrient and oxygen supply, a condition that may predispose it to imbalances between oxygen delivery and metabolic waste removal [11]. This high metabolic state enhances ROS generation via the mitochondrial electron transport chain, and with aging or external stressors, the decline in antioxidant defenses facilitates ROS accumulation [4].

Chronic light exposure is another major contributor to macular vulnerability. Short-wavelength visible light in the blue–violet range induces photosensitization within the retina, generating ROS through photochemical reactions. Among the most studied photosensitizing molecules are bisretinoids, such as N-retinylidene-N-retinylethanolamine (A2E), which accumulate in the lipofuscin of RPE cells. Classic experimental work has demonstrated that A2E is highly phototoxic under blue light, inducing RPE apoptosis and mitochondrial dysfunction [12]. Spectral analyses in A2E-containing RPE cells have shown that the 415–455 nm band generates the highest ROS levels and induces significant mitochondrial impairment [13]. These findings provide a physiological and therapeutic rationale for the protective roles of optical filters and macular pigments such as lutein and zeaxanthin.

The macular region is also rich in PUFAs. Photoreceptor outer segments contain abundant PUFAs, and the RPE phagocytoses and processes these segments daily, subjecting it to a constant oxidative load from lipid metabolism [4]. With aging or impairment of lysosomal/autophagic function, incompletely degraded lipids accumulate, leading to the formation of lipofuscin and sub-RPE deposits known as drusen. Drusen are lipid-rich, and local lipid peroxidation can trigger chronic inflammation, activate the complement system and immune pathways, and accelerate RPE and photoreceptor dysfunction [4,11].

Another core aspect of vulnerability is mitochondrial dysfunction. Both RPE cells and photoreceptors have high ATP requirements, with mitochondria serving as the principal source of both energy and ROS. Aging and environmental stressors—including cigarette smoke, ultraviolet (UV) and visible light exposure, and high-fat diets—can cause mitochondrial deoxyribonucleic acid [DNA] (mtDNA) damage, impair respiratory chain efficiency, and disrupt mitochondrial dynamics (fusion/fission) and mitophagy. These alterations increase ROS production and diminish antioxidant responses, promoting RPE senescence and cell death [14]. Mitochondria-derived ROS and lipid peroxidation can exacerbate each other, leading to lysosomal and autophagic dysfunction, oxidative modification of membrane proteins, and a self-perpetuating cycle of intracellular oxidative injury.

**Figure 2:**
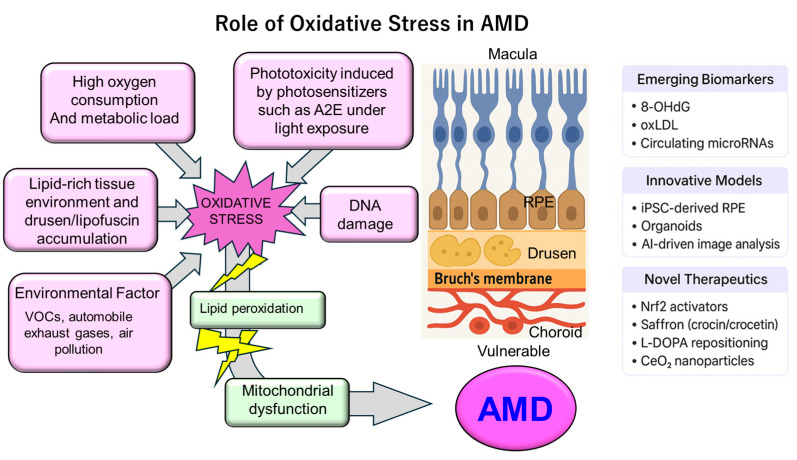
Schematic representation of oxidative stress in the pathogenesis of AMD. The macula is highly susceptible to oxidative injury due to elevated oxygen consumption, intense metabolic activity, and phototoxicity from chronic light exposure, particularly short-wavelength (blue) light, mediated by photosensitizers such as A2E. Environmental insults, including cigarette smoke and VOCs, further drive lipid peroxidation and impair redox balance. The lipid-rich retinal environment, coupled with drusen and lipofuscin accumulation, exacerbates oxidative stress and mitochondrial dysfunction in the RPE, photoreceptors, and Bruch’s membrane. These events interact with inflammatory and complement pathways, ultimately promoting AMD progression. 8-OHdG = 8-hydroxy-2′-deoxyguanosine, A2E = N-retinylidene-N-retinylethanolamine, AI = Artificial intelligence, AMD = Age-related macular degeneration, CeO_2_ = Cerium oxide, iPSC = Induced pluripotent stem cell, L-DOPA = Levodopa, Nrf2 = Nuclear factor erythroid 2-related factor 2, oxLDL = Oxidized low-density lipoprotein, RNA = Ribonucleic acid, RPE = Retinal pigment epithelium, VOC = Volatile organic compound.

As illustrated in Figure 2, the macula’s susceptibility to oxidative stress results from the combined effects of: (1) high oxygen consumption and metabolic demand; (2) phototoxicity from chronic light exposure—particularly blue-light sensitivity mediated by photosensitizers such as A2E; (3) a lipid-rich environment prone to drusen and lipofuscin accumulation; and (4) mitochondrial dysfunction. These insights underscore the central role of oxidative stress in AMD pathogenesis and provide a theoretical foundation for interventions such as antioxidant nutritional therapy (e.g., the Age-Related Eye Disease Study (AREDS) supplements), wavelength-selective light filtering, and novel treatments aimed at improving mitochondrial function [4,11,12,13,14].

While the roles of A2E photosensitization, PUFA oxidation, and mitochondrial dysfunction are well established, recent studies highlight several areas of conflicting evidence and emerging mechanisms that require further investigation. For example, although optical blue-light filtering has been proposed as a protective strategy against A2E-mediated phototoxicity, large-scale clinical trials have failed to demonstrate consistent benefits in reducing AMD incidence or progression [15]. This discrepancy suggests that other mechanisms beyond simple light absorption may predominate in vivo.

Similarly, the contribution of PUFA oxidation to AMD pathogenesis remains debated. Epidemiological studies have reported protective associations between dietary omega-3 intake and AMD progression, yet randomized clinical trials (e.g., AREDS2) did not confirm significant benefits [16]. This paradox underscores the need to clarify whether PUFA oxidation is primarily pathogenic or if certain lipid metabolites may exert protective effects depending on context.

Emerging evidence also suggests that ferroptosis, a lipid peroxidation–driven form of regulated cell death, may play a critical role in RPE degeneration under chronic oxidative stress [17]. In addition, alterations in mitochondrial dynamics, including imbalances in fission and fusion or impaired mitophagy, have been identified as key determinants of cellular susceptibility to oxidative damage [18]. These novel mechanistic insights provide testable hypotheses for future studies and highlight the complexity of oxidative vulnerability in the macula beyond classical paradigms.

## 3. Sources of Oxidative Stress in AMD

**Figure 3:**
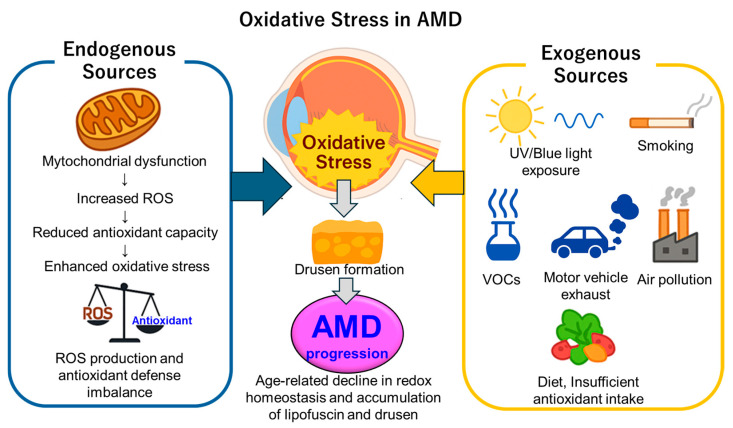
Endogenous and exogenous sources of oxidative stress in AMD. Schematic illustration summarizing the major internal and external drivers of oxidative stress in AMD. Endogenous sources include mitochondrial dysfunction, excessive ROS production, impaired antioxidant defenses, lipid peroxidation, and chronic inflammation within the RPE and photoreceptors. Exogenous sources include cigarette smoke, VOCs, motor vehicle exhaust, blue light and UV radiation, dietary deficiencies in antioxidants, and environmental pollutants. These combined factors accelerate oxidative damage to retinal cells, promote drusen formation, and activate inflammatory and complement pathways, thereby contributing to the onset and progression of AMD. AMD = Age-related macular degeneration, ROS = Reactive oxygen species, UV = Ultraviolet, VOC = Volatile organic compound.

Oxidative stress plays a central role in the pathogenesis of AMD and can be broadly categorized into endogenous sources (arising within retinal tissues) and exogenous sources (stemming from environmental and lifestyle factors) (Figure 3). This chapter first outlines the intrinsic sources within the RPE and photoreceptors, followed by the contributions of light exposure, smoking, air pollution, and dietary patterns.

### 3.1. Endogenous Factors

#### 3.1.1. Mitochondrial Dysfunction in RPE and Photoreceptors

RPE cells and photoreceptors exhibit exceptionally high metabolic demands, producing large quantities of ATP via mitochondrial oxidative phosphorylation. Mitochondria are the principal sites of ROS generation as by-products of the electron transport chain. Aging, genetic susceptibility, and oxidative insults can induce mtDNA damage and impair respiratory chain efficiency, leading to excess ROS production and cellular injury [19,20]. Histopathological and ultrastructural studies of AMD donor eyes have consistently documented reduced mitochondrial number, morphological abnormalities, and impaired respiratory capacity in RPE [19]. These findings strongly suggest a critical association between mitochondrial dysfunction and AMD pathogenesis; however, whether mitochondrial damage acts as a primary driver or arises as a consequence of disease progression remains unresolved. Thus, mitochondrial impairment should be considered a central hypothesis in AMD research, warranting further mechanistic and longitudinal studies.

#### 3.1.2. Imbalance Between ROS Production and Antioxidant Defense

Cells employ enzymatic antioxidant systems—including superoxide dismutases (SODs), catalase, and the γ-glutamyl-cysteinyl-glycine (glutathione: GSH) system—to neutralize ROS. However, with aging and chronic stress, the activity of these defense mechanisms declines. The transcription factor nuclear factor erythroid 2-related factor 2 (Nrf2, gene name NFE2L2) serves as a master regulator of antioxidant gene expression, and reduced Nrf2 responsiveness increases RPE susceptibility to oxidative injury [21]. Impairment of the Nrf2 pathway, along with regulators of mitochondrial biogenesis and proteostasis such as PGC-1α, leads to ROS accumulation and insufficient repair capacity [21].

#### 3.1.3. Age-Related Decline in Redox Homeostasis and Accumulation of Oxidized Metabolites

With aging, oxidatively modified by-products such as lipofuscin accumulate within RPE cells. Lipofuscin is inherently photoreactive, generating ROS upon light exposure. Declines in photoreceptor outer segment turnover efficiency and lysosomal function contribute to the build-up of incompletely degraded material, including lipofuscin and drusen. These deposits provoke local inflammation and activate the complement cascade, establishing a feedback loop that exacerbates oxidative stress [19,20,22,23].

These endogenous factors are highly interconnected, forming a pathogenic cycle: mitochondrial impairment → increased ROS → lysosomal/autophagic dysfunction → accumulation of lipofuscin/drusen → inflammation and further oxidative stress. This vicious cycle is considered a central mechanism in dry AMD and geographic atrophy [19,20].

### 3.2. Exogenous Factors

#### 3.2.1. Light Exposure (Phototoxicity)

The retina is chronically exposed to visible light, with short-wavelength blue light being particularly effective in inducing ROS through photochemical reactions. Bisretinoids such as A2E, a major component of lipofuscin in RPE cells, act as potent photosensitizers under blue-light illumination, triggering oxidative damage and apoptosis in RPE [12]. Recent work suggests that A2E in combination with blue light may disrupt iron metabolism and the GSH peroxidase 4 (GPX4) pathway, potentially inducing a ferroptosis-like lipid peroxidation–dependent form of cell death [24].

#### 3.2.2. Smoking

Smoking is one of the strongest modifiable risk factors for AMD, with robust epidemiological evidence supporting its association [25]. Cigarette smoke delivers oxidants directly and depletes systemic antioxidant reserves such as vitamin C, thereby increasing oxidative burden on the retina and RPE. Smoking also impacts ocular perfusion, immune responses, and complement activation, contributing to both the onset and progression of AMD.

#### 3.2.3. Air Pollution Including VOCs

Recent epidemiological meta-analyses and large-scale cohort studies suggest positive correlations between exposure to air pollutants—such as particulate matter with an aerodynamic diameter of less than 2.5 μm (PM_2.5_), nitrogen dioxide (NO_2_), and ozone (O_3_). These pollutants enter systemic circulation via inhalation and can provoke oxidative stress and inflammation, potentially affecting the retina. VOCs are of particular interest because their metabolism may yield reactive oxidizing intermediates, posing a chronic oxidative load to retinal tissues. However, quantitative exposure assessment and causal inference remain active areas of investigation [26].

While epidemiological studies consistently suggest an association between ambient air pollutants and AMD risk, the exposure–response relationship remains complex and not fully elucidated. For example, PM_2.5_ and NO_2_ exposure have been linked to retinal thinning and AMD prevalence, but confounding factors such as smoking, occupational exposures, and genetic susceptibility complicate causal inference [27]. Furthermore, VOCs represent a particularly underexplored category; their biotransformation generates reactive intermediates that may accumulate in the retina, yet dose–response thresholds and synergistic interactions with other pollutants remain undefined [28]. Future work should integrate personalized exposure monitoring, omics-based biomarkers, and gene–environment interaction studies to disentangle these complex pathways.

#### 3.2.4. Diet and Oxidative Load

Dietary patterns strongly influence redox balance. Diets high in saturated fats and processed foods elevate oxidative burden, whereas nutrients with antioxidant and anti-inflammatory properties—such as lutein, zeaxanthin, vitamins C and E, zinc, and omega-3 fatty acids—are associated with slower AMD progression in clinical trials, including AREDS and AREDS2 [16,29]. These findings suggest protective effects mediated, at least in part, through the attenuation of oxidative stress. Nonetheless, the influence of individual variability (e.g., genetic background) and the optimization of nutrient dose and composition remain unresolved.

Beyond conventional antioxidant nutrients, saffron-derived compounds such as crocin, crocetin, and safranal have emerged as promising dietary factors with antioxidant, anti-inflammatory, and neuroprotective properties. Randomized controlled trials have reported improvements in retinal function and visual acuity among AMD patients receiving saffron supplementation [30]. Mechanistically, crocetin has been shown to modulate mitochondrial function and attenuate ROS generation in RPE models [31]. These findings suggest that saffron-derived molecules may complement conventional antioxidant therapies, though long-term efficacy, optimal dosing, and interaction with genetic background remain to be clarified.

## 4. Molecular Mechanisms Linking Oxidative Stress to AMD Pathogenesis

The pathogenesis of AMD progresses through complex molecular mechanisms centered on oxidative stress, which act on the RPE and photoreceptors. The major mechanisms are described below.

### 4.1. Oxidative Damage in RPE Cells

The RPE performs diverse, energy-intensive functions, including light absorption, phagocytosis of photoreceptor outer segments, and vitamin A metabolism, all of which require efficient mitochondrial ATP production. Mitochondrial dysfunction or inefficiency in the electron transport chain results in excessive generation of ROS, such as superoxide and hydrogen peroxide, leading to oxidative damage to proteins, lipids, and DNA. Age-related or stress-induced declines in oxidative repair systems and lysosomal/autophagic pathways in the RPE cause cumulative oxidative injury, ultimately leading to cellular dysfunction and death via apoptosis, necrosis, or ferroptosis-like pathways [4,19].

### 4.2. Lipid Peroxidation and Advanced Lipoxidation End Products (ALEs)

The macula and photoreceptor outer segments are rich in PUFAs, rendering them highly susceptible to lipid peroxidation. Reactive carbonyl species generated during lipid peroxidation (e.g., 4-hydroxy-2-nonenal [4-HNE], malondialdehyde [MDA]) form covalent adducts with proteins, yielding ALEs. These ALEs not only induce protein cross-linking and denaturation but also activate inflammatory signaling through receptors such as RAGE (receptor for advanced glycation end products (AGEs) and ALEs)). Accumulation of ALEs and AGEs disrupts intracellular signaling and reinforces the oxidative stress–inflammation cycle [32,33].

### 4.3. Drusen Formation and Complement Activation

Drusen, which accumulate at the RPE–Bruch’s membrane interface, are lipid- and protein-rich deposits containing complement components. The identification of complement fragments (C3, C5b-9, etc.) within drusen, together with the association of complement-related gene polymorphisms (CFH, C3, C2/complement factor B: CFB) with AMD risk, supports a role for dysregulated complement activation in drusen formation and inflammation [34,35]. Oxidatively modified proteins and ALEs stimulate complement and immune receptors, amplifying the complement cascade and linking oxidative stress to complement overactivation [9,35].

### 4.4. Crosstalk Among Oxidative Stress, Inflammation, and Autophagy

Oxidative stress activates inflammatory signaling pathways such as the nuclear factor kappa B (NF-κB) and inflammasomes, promoting the production of pro-inflammatory cytokines. Autophagy–lysosomal pathways are critical for removing oxidatively damaged components; when impaired, lipofuscin and dysfunctional mitochondria accumulate, further enhancing ROS production. Conversely, excessive oxidative stress can inhibit autophagic function, creating a positive feedback loop that exacerbates chronic inflammation and cell death in AMD [36,37,38,39]. Recent evidence suggests that oxidative stress modulates mitophagy, which helps maintain RPE homeostasis, and that mitophagy impairment may contribute to the development of geographic atrophy [37,40].

### 4.5. Nrf2 Pathway Dysregulation

NFE2L2 is a master transcription factor regulating antioxidant defense by inducing enzymes such as SOD, GSH S-transferase (GST), heme oxygenase-1 (HO-1), and GSH synthesis-related genes. Reduced Nrf2 activity in the RPE directly contributes to oxidative damage accumulation and functional decline. Aging, genetic factors, and environmental stressors have all been shown to suppress Nrf2 signaling, leading to depletion of antioxidant capacity. Restoration of Nrf2 activity is thus considered a promising therapeutic approach for AMD [21,41,42].

### 4.6. Pathway Crosstalk and Emerging Multi-Target Therapeutic Strategies

Recent evidence suggests that mitochondrial dysfunction, lipid peroxidation, and complement activation should not be viewed as isolated processes but as interconnected elements of a pathogenic network. For example, lipid peroxidation products such as 4-HNE not only damage proteins and DNA but also act as danger-associated molecular patterns (DAMPs) that activate complement and toll-like receptors, thereby linking oxidative injury to chronic inflammation [43]. Similarly, mitochondrial dysfunction enhances ROS production, which promotes both ferroptosis and complement deposition in the RPE, reinforcing tissue degeneration [44].

From a therapeutic standpoint, these insights suggest that interventions targeting a single pathway may be insufficient. Instead, multi-target approaches are being explored, such as combined activation of Nrf2 with concurrent inhibition of complement or ferroptosis pathways [45]. Moreover, immunometabolic reprogramming of microglia and macrophages has emerged as a potential strategy to attenuate the self-amplifying oxidative stress–inflammation cycle [46]. Together, these findings point toward integrative and combinatorial therapies that address the cross-talk among oxidative stress, inflammation, and immune dysregulation rather than isolated downstream events.

### 4.7. Microglia and Macrophage Contributions to AMD Pathogenesis

Beyond the RPE, innate immune cells such as microglia and macrophages play critical roles in the development and progression of AMD. Activated microglia migrate from the inner retina to the subretinal space, where they secrete pro-inflammatory cytokines, complement components, and ROS, thereby amplifying local oxidative stress and tissue injury [47,48]. Macrophages infiltrating from the choroid can exert dual roles: under low-to-moderate ROS levels, they contribute to debris clearance and homeostatic maintenance, whereas sustained high ROS exposure drives polarization toward pro-inflammatory phenotypes that promote photoreceptor and RPE degeneration [49,50]. These observations highlight that ROS act not only as damaging mediators but also as physiological signaling molecules, with outcomes depending on their concentration, duration of exposure, and the cellular context [48]. This bidirectional effect underscores the importance of redox balance in retinal homeostasis and suggests that therapeutic modulation of microglial and macrophage activity may represent a promising strategy in AMD.

### 4.8. Dose-Dependent Effects of ROS on AMD Progression

Emerging evidence suggests that the effects of ROS on RPE and photoreceptor cells may be dose-dependent. While low to moderate ROS levels can activate adaptive cellular responses and antioxidant defense mechanisms, high ROS levels can cause oxidative damage, mitochondrial dysfunction, and cell death, potentially accelerating AMD progression. This dual role underscores the importance of considering ROS intensity in both mechanistic studies and therapeutic strategies [43,51].

### 4.9. Integrative Perspective

Collectively, oxidative stress in AMD pathogenesis does not result from a single toxic molecule but rather emerges from the interplay of multiple pathways:

(1) metabolic and mitochondrial vulnerability in the RPE;
(2) lipid peroxidation and ALE/AGE accumulation;
(3) complement and immune activation driven by drusen;
(4) impairment of autophagy, mitophagy, and lysosomal function;
(5) downregulation of antioxidant gene networks such as Nrf2.

These pathways form a self-amplifying oxidative stress–inflammation–cell death loop that drives AMD onset and progression. Understanding these mechanisms provides the basis for diverse therapeutic strategies, including complement inhibition, activation of antioxidant responses, modulation of lipid metabolism, and restoration of autophagic or lysosomal function. Recent research supports the need for multi-target approaches that address several of these pathways simultaneously [52].

## 5. Clinical Evidence on Oxidative Stress

### 5.1. Oxidative Damage Markers in Serum, Aqueous Humor, and Retinal Tissue

In patients with AMD, serum levels of oxidative stress markers such as MDA, protein carbonyls, and 8-hydroxy-2′-deoxyguanosine (8-OHdG) are significantly elevated compared with controls, indicating increased systemic oxidative burden [6]. In the aqueous humor, marked reductions in the anti-aging protein Klotho have been reported alongside elevated levels of 8-OHdG and the pro-inflammatory cytokine Interleukin (IL)-6, coupled with decreased total antioxidant status (TAS) and anti-inflammatory IL-10. These findings highlight their potential as clinical markers of intraocular oxidative stress and inflammation [53]. Furthermore, the activities of antioxidant enzymes such as SOD, catalase, and GSH peroxidase are reduced in both serum and aqueous humor of AMD patients compared with healthy individuals, suggesting impaired antioxidant defense mechanisms [21].

In studies on glaucoma, markers of oxidative protein damage (protein carbonyls), as well as AGEs and ALEs, have been shown to be significantly elevated in serum and aqueous humor (e.g., serum: 584 vs. 1085 nmoL/mg; aqueous humor: 8.8 vs. 23 nmoL/mg) [54]. Although these findings are derived from glaucoma research, they may also be applicable to elucidating intraocular oxidative stress in AMD, warranting further investigation.

### 5.2. Imaging Findings Reflecting Oxidative Damage

Currently, there are no diagnostic techniques capable of directly visualizing oxidative stress in vivo. However, optical coherence tomography (OCT) findings such as the presence of drusen, morphological alterations of the RPE, and abnormalities in retinal thickness and architecture are considered indirect indicators of oxidative degeneration [55]. In addition, ongoing proteomic analyses of aqueous humor have revealed changes in the expression of proteins linked to inflammation and oxidative stress, such as secreted protein, acidic and rich in cysteine (SPARC) related modular calcium binding 2 (SMOC2) and IL-6 [56]. These approaches may evolve into noninvasive techniques for detecting and monitoring oxidative stress-related biomarkers, including oxidatively modified proteins, within the eye.

### 5.3. Oxidative Stress–Related Genetic Polymorphisms and Susceptibility

Among genetic factors in AMD, polymorphisms in complement pathway genes—most notably CFH Y402H—are well-established and have also been implicated in oxidative stress-related susceptibility [57,58]. The CFH risk allele (Y402H) has reduced binding affinity for oxidatively modified epitopes, such as oxidized low-density lipoprotein (oxLDL), thereby impairing complement-mediated antioxidant protection and increasing susceptibility to AMD [57].

Regarding oxLDL and homocysteine, a case–control study comparing 45 patients with exudative AMD and 45 age- and sex-matched healthy controls found a significant positive correlation between plasma oxLDL levels and homocysteine concentrations in the exudative AMD group [59]. In contrast, a prospective cohort study involving 2468 participants with a 25-year follow-up found no association between serum oxLDL cholesterol levels and AMD progression or late-stage AMD incidence [60]. Similarly, a smaller-scale study reported no significant correlation between serum oxLDL levels and AMD status [61]. These discrepancies between short-term and long-term studies highlight conflicting evidence regarding the contribution of oxLDL and homocysteine to AMD onset and progression, underscoring the need to clarify the interplay between genetic predisposition and environmental/metabolic factors.

Other studies have linked AMD risk to polymorphisms in genes related to oxidative stress responses and DNA repair [62]. For example, the succinate receptor 1 (SUCNR1) receptor polymorphism rs13315275 has been associated with increased risk of late-stage dry AMD [63], and elevated aqueous succinate levels have also been observed in AMD patients [63], suggesting a role for metabolic signaling in AMD pathogenesis.

### 5.4. Conflicting Evidence on Oxidative Stress

Conflicting evidence remains regarding the role of oxidative biomarkers such as oxLDL and homocysteine in AMD pathogenesis. For example, case–control studies have reported significantly higher plasma oxLDL and homocysteine levels in patients with exudative AMD compared with controls, suggesting a potential pathogenic link [59]. In contrast, large-scale longitudinal studies have failed to confirm these associations, showing no significant relationship between baseline oxLDL levels and the risk of progression to late AMD [64,65]. These discrepancies may reflect methodological differences, such as sample size, follow-up duration, and population genetics. They also suggest that oxidative biomarkers alone may not sufficiently predict AMD risk and must be interpreted within the broader context of genetic susceptibility (e.g., CFH Y402H polymorphism) and environmental exposures.

This inconsistency underscores a critical knowledge gap: whether oxidative biomarkers represent causal mediators of AMD or merely epiphenomena of disease progression. Future studies should employ standardized biomarker panels, longitudinal sampling, and integrative genomic analyses to disentangle causal pathways. A testable hypothesis is that specific genotypes (e.g., CFH, ARMS2/HTRA1 variants) may modulate individual oxidative stress responses to environmental exposures such as smoking and diet, thereby influencing biomarker expression and AMD risk [57].

### 5.5. Emerging Biomarkers and Personalized Therapeutic Implications

Recent advances have identified novel biomarkers beyond conventional oxidative stress markers, offering opportunities for personalized therapeutic strategies in AMD. For instance, proteomics of aqueous humor has revealed differentially expressed proteins—such as apolipoprotein A1 (APOA1), serotransferrin (TF), complement C3, and lipocalin-1 (LCN1)—which were identified by a meta-analysis of mass spectrometry studies using ocular fluids and may serve as systemic or local indicators of disease state and progression [66]. In parallel, metabolomics studies have identified altered lipid and lipoprotein metabolism (e.g., changes in large and extra-large high-density lipoprotein (HDL) particles) associated with AMD, which correlate with genetic risk loci and may modify response to antioxidant therapies [67].

From a genetic standpoint, the integration of genetic profiling with oxidative stress biomarkers is gaining traction. Variants in CFH and ARMS2/HTRA1 not only modulate AMD susceptibility but appear to influence downstream oxidative stress responses to environmental exposures such as smoking and diet, as shown in nutritional genomics studies [68]. For example, a study found that minor alleles in lipid metabolism genes, such as apolipoprotein E (APOE) and hepatic lipase (LIPC), are associated with both AMD risk and with differential lipid biomarker signatures, suggesting that biomarker–genetic panels could stratify patients more effectively for antioxidant or complement-targeted therapies.

### 5.6. Summary

Clinical evidence strongly supports the involvement of oxidative stress in AMD pathogenesis from multiple perspectives. Elevated systemic and intraocular oxidative stress markers—including serum MDA, 8-OHdG, protein carbonyls, and aqueous humor Klotho, TAS, IL-6, and IL-10—indicate increased oxidative burden. OCT-detected structural abnormalities and proteomic markers such as SMOC2 and IL-6 offer indirect but valuable indicators of oxidative injury. Moreover, oxidative stress-related genetic polymorphisms, such as CFH Y402H, and metabolic products such as oxLDL, may influence AMD susceptibility and progression. Future research should focus on integrating these biomarkers into predictive models for diagnosis and prognosis, with potential applications in personalized medicine.

## 6. Therapeutic Strategies Targeting Oxidative Stress in AMD

**Figure 4:**
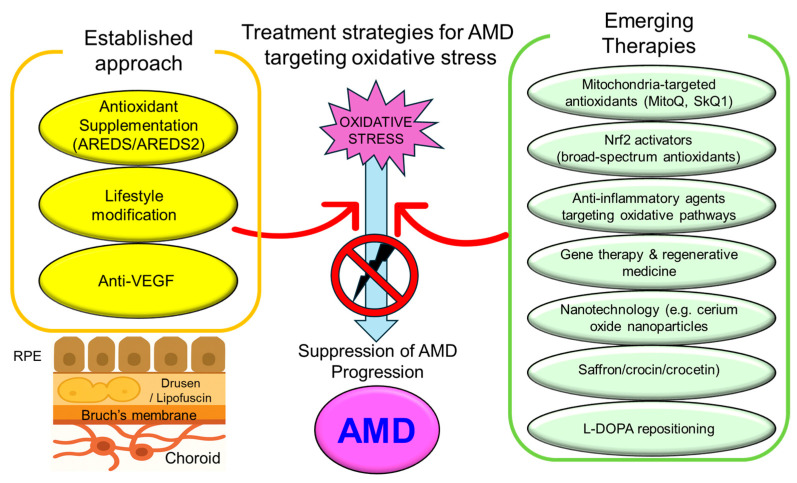
Schematic overview of established and emerging therapeutic strategies targeting oxidative stress in AMD. The figure highlights two main categories of interventions aimed at counteracting oxidative stress, a key driver in AMD pathogenesis. On the left, established approaches include antioxidant supplementation (based on AREDS and AREDS2 formulations), lifestyle modification, and anti-VEGF therapy. On the right, emerging therapies involve mitochondrial-targeted antioxidants (e.g., Mitoquinone: MitoQ; Skulachev Ion-1: SkQ1), Nrf2 activators (broad-spectrum antioxidants), anti-inflammatory agents targeting oxidative pathways, gene therapy and regenerative medicine, nanotechnology-based treatments (e.g., cerium oxide nanoparticles), dietary components (saffron, crocin, crocetin), and Levodopa (L-DOPA) repositioning. These strategies collectively aim to reduce ROS, restore redox homeostasis, and ultimately slow or prevent AMD progression. AMD = Age-related macular degeneration, AREDS = Age-Related Eye Disease Study, L-DOPA = Levodopa, MitoQ = Mitoquinone, Nrf2 = Nuclear factor erythroid 2-related factor 2, RPE = Retinal pigment epithelium, SkQ1 = Skulachev Ion-1.

Given the central role of oxidative stress in the pathogenesis of AMD, therapeutic interventions targeting oxidative pathways have garnered significant attention. These interventions can be broadly categorized into “established approaches” and “emerging therapies”, as summarized in Figure 4.

### 6.1. Established Approaches

#### Antioxidant Supplementation Therapy (AREDS/AREDS2 Trials)

The AREDS demonstrated that high-dose antioxidant vitamins (vitamins C, E, β-carotene) combined with zinc supplementation could reduce the progression of intermediate or advanced AMD by up to 25% [69]. The follow-up AREDS2 trial modified the formulation by replacing β-carotene with lutein and zeaxanthin, and by attempting the addition of omega-3 fatty acids [16]. Due to the increased risk of lung cancer in smokers associated with β-carotene, its exclusion has been recommended, thereby improving both safety and efficacy [70]. However, the addition of omega-3 fatty acids (docosahexaenoic acid: DHA and eicosapentaenoic acid: EPA) did not show any significant additional benefit in preventing AMD progression [70].

### 6.2. Emerging Therapeutic Trends

#### 6.2.1. Mitochondria-Targeted Antioxidants

Mitochondria-targeted antioxidants such as MitoQ and SkQ1 have emerged as promising candidates. MitoQ, a ubiquinone derivative conjugated with the lipophilic cation triphenylphosphonium (TPP^+^), accumulates in mitochondria and effectively scavenges ROS, showing therapeutic potential [3]. SkQ1, a plastoquinone-like compound, has demonstrated protective effects against AMD-like lesions in aged AMD models (e.g., oxidative stress-prone: OXYS rats) when administered via eye drops or orally. It also reduces amyloid-β accumulation and mammalian target of rapamycin (mTOR) signaling [71].

#### 6.2.2. Nrf2 Activators (e.g., Bardoxolone Methyl, Sulforaphane)

The Nrf2 pathway, a key regulator of the antioxidant response, is a promising therapeutic target for enhancing cellular defense mechanisms. Sulforaphane, a compound derived from broccoli, is a potent Nrf2 activator that induces a broad range of antioxidant genes [72]. Bardoxolone methyl (CDDO-Me, the methyl ester of 2-cyano-3,12-dioxooleana-1,9(11)-dien-28-oic acid), a synthetic triterpenoid, also activates Nrf2 and modulates both Nrf2 and NF-κB pathways. Although its dual action is promising, concerns remain regarding its cardiovascular side effects, and further safety evaluations are warranted [73,74].

#### 6.2.3. Anti-Inflammatory Agents Modulating Oxidative Pathways

Oxidative stress and inflammation are closely intertwined. Agents targeting not only Nrf2 but also NF-κB and inflammasome pathways may disrupt the oxidative stress–inflammation loop. Although such agents (e.g., CDDO-Me) are currently at the preclinical stage, they represent a promising direction for future clinical development [74].

#### 6.2.4. Gene Therapy and Regenerative Strategies

To fundamentally counteract oxidative stress, novel approaches such as gene therapy (e.g., Nrf2 gene delivery) and regenerative medicine using stem cells for RPE regeneration are under active investigation. However, these strategies remain primarily preclinical, and further studies are needed to establish their long-term safety and efficacy.

#### 6.2.5. Saffron and Its Carotenoid Constituents

Saffron (Crocus sativus) and its bioactive carotenoids (crocin, crocetin) have attracted attention as antioxidant and neuroprotective agents for retinal disease [75,76,77,78]. Several robust preclinical studies in animal models demonstrate that saffron and its constituents protect retinal structure and function. For example, oral saffron supplementation preserved photoreceptor morphology and retinal function in a light-induced retinal damage model in rats (Maccarone et al., IOVS 2008) [79]. Crocetin has been shown to reduce retinal cell death in ischemia/reperfusion and excitotoxic models, with modulation of caspase activity and oxidative stress pathways (Heydari et al., 2023) [80]. Additionally, saffron extracts (standardized for crocin content) reduced microglial activation and prevented retinal ganglion cell loss in a mouse ocular-hypertension model, indicating broader neuroprotective and anti-inflammatory actions beyond photoreceptors. Recent in vitro studies further support crocin’s ability to mitigate photoreceptor oxidative/apoptotic injury. These preclinical data support mechanistic bases—antioxidant, anti-apoptotic, anti-inflammatory, and mitochondrial protective effects—for the clinical improvements reported in small randomized trials and longitudinal studies in early or mild–moderate AMD (improvements in flicker sensitivity, ERG parameters, contrast sensitivity, and modest visual acuity gains after short-to-mid-term supplementation). Importantly, several randomized clinical trials and longitudinal studies in patients with early or mild–moderate AMD have reported functional benefits—including improved retinal flicker sensitivity, electroretinogram parameters, contrast sensitivity, and modest gains in visual acuity—after short- to mid-term oral supplementation (typical doses 20–30 mg saffron or 5–15 mg crocin daily, treatment 3–12 months) [75,76,77,78]. While encouraging, the preclinical and clinical studies are heterogeneous in models, dose, and endpoints; therefore larger, longer randomized clinical trials with standardized outcomes are still required to confirm efficacy and guide clinical recommendations [75,76,77,78].

#### 6.2.6. L-DOPA Repositioning in AMD Prevention and Treatment

In addition to conventional antioxidant supplementation and novel mitochondrial-targeted therapies, repurposing of existing drugs has gained attention as a promising approach. Notably, L-DOPA, a mainstay therapy for Parkinson’s disease, has been associated with a reduced risk of AMD development and delayed disease onset in epidemiological studies [81]. L-DOPA serves as a precursor in melanin synthesis within the RPE and exhibits antioxidant as well as cytoprotective effects [82]. Mechanistically, L-DOPA may enhance RPE resilience against oxidative stress and modulate signaling pathways relevant to retinal health [83]. Clinical evidence suggests that patients receiving L-DOPA therapy for Parkinson’s disease show a lower incidence and severity of AMD compared with untreated populations [81]. These findings highlight L-DOPA repositioning as an innovative therapeutic avenue warranting further investigation in AMD.

#### 6.2.7. Cerium Oxide Nanoparticles (CeO_2_-NPs) and Retinal Protection

Recent advances in nanotechnology have highlighted CeO_2_-NPs (nanoceria) as potent catalytic antioxidants with a unique self-regenerative redox capacity, arising from the cycling between Ce^3+^ and Ce^4+^ states on their surface. Preclinical studies demonstrated that intravitreal administration of CeO_2_-NPs preserves retinal morphology and function, prevents photoreceptor degeneration, and attenuates oxidative stress–induced damage in animal models of retinal degeneration [84]. Moreover, CeO_2_-NPs have been shown to inhibit drusen-like deposits and modulate inflammatory signaling pathways, thereby providing both neuroprotective and anti-inflammatory benefits [85,86,87]. For example, Fiorani et al. showed that intravitreal injection of nanoceria prior to light exposure maintained outer nuclear layer thickness, reduced photoreceptor apoptosis, and inhibited microglial activation, with nanoparticles persisting in the photoreceptor outer segments for weeks (Fiorani et al., *PLoS ONE*, 2015) [88]. Beyond these observations, Maccarone et al. comprehensively reviewed the ophthalmic applications of CeO_2_-NPs, emphasizing their long-lasting neuroprotective effects, ability to modulate inflammatory signaling pathways, and promising formulation strategies such as PEGylation, liposomal encapsulation, and chitosan coatings to improve stability and ocular delivery (Maccarone et al., *J. Ocul. Pharmacol. Ther.*, 2020) [89]. These studies indicate that nanoceria not only scavenge reactive oxygen species by mimicking endogenous antioxidant enzymes such as superoxide dismutase and catalase, but also downregulate pro-inflammatory mediators and microglial reactivity, thereby providing both antioxidative and anti-inflammatory benefits (Dhall and Self, Nanomaterials 2018) [90]. Importantly, particle size, surface chemistry, and Ce^3+^/Ce^4+^ ratio influence their biological activity, bioavailability, and safety profile, and while most preclinical studies report favorable tolerability, long-term clearance, potential aggregation, and effects in larger animal models remain insufficiently characterized (Corsi et al., 2023) [91]. Overall, CeO_2_-NPs represent a promising disease-modifying nanomedicine for AMD that extends beyond conventional antioxidant supplementation and anti-VEGF therapy, yet further studies are required to clarify optimal formulations, therapeutic timing, and translational safety to fully harness their regenerative and neuroprotective potential.

#### 6.2.8. Limitations of AREDS and Personalized Responses

The AREDS and its follow-up AREDS2 demonstrated that high-dose antioxidant and zinc supplementation can reduce the risk of progression to advanced AMD in individuals with intermediate disease, establishing the basis for current clinical recommendations [16]. However, these landmark trials also have important limitations. First, treatment benefit was not universal: patients with early AMD or advanced bilateral disease derived little measurable improvement, suggesting stage-dependent efficacy. Second, inter-individual variability in response has been increasingly recognized, driven by genetic background, smoking status, and baseline nutritional status. In particular, pharmacogenetic studies have reported that *CFH* and *ARMS2/HTRA1* polymorphisms influence outcomes, with some genotypes showing reduced or even adverse responses to zinc supplementation [92,93]. These findings highlight that AREDS supplementation, while effective in select populations, is not a “one-size-fits-all” intervention. Moving forward, the integration of genetic profiling, metabolomic markers, and clinical phenotyping is essential to refine patient selection and optimize dosing strategies. Such precision-nutrition approaches may ultimately transform antioxidant therapy from a generalized recommendation into a personalized preventive intervention in AMD.

### 6.3. Summary

Antioxidant supplementation therapy based on the AREDS trials remains the standard clinical approach for intermediate-stage AMD. The AREDS2 formulation further improved upon the original in terms of safety and effectiveness. Looking ahead, mitochondria-targeted antioxidants and Nrf2 activators represent mechanism-based therapies that may form the basis of personalized medicine in AMD. Additionally, addressing oxidative-inflammatory crosstalk and cellular recycling pathways such as autophagy offers a promising multilayered strategy. Ultimately, an integrated multimodal treatment paradigm, combining these approaches, may be key to preventing or halting AMD progression in the future.

To provide a more systematic overview of current and emerging therapeutic strategies, we have included a comparative summary table (Table 1). This table contrasts established interventions such as AREDS-based supplementation and anti-VEGF therapy with novel approaches including saffron, L-DOPA, nanotechnology-based antioxidants, and Nrf2 activators. By outlining their mechanisms of action, levels of clinical evidence, and stages of development, the table highlights both the robustness of established therapies and the promise of innovative strategies that may shape future AMD management. This integrative perspective emphasizes the dynamic and evolving therapeutic landscape, thereby offering clinicians and researchers a clearer framework for translating oxidative stress–targeted interventions into clinical practice.

## 7. Challenges and Future Perspectives

Therapeutic strategies targeting oxidative stress in AMD include both established and emerging approaches, each with distinct challenges and opportunities for advancement. In this section, we outline three key areas for future consideration.

### 7.1. Limitations of Conventional Antioxidant Therapies

The antioxidant supplementation strategies evaluated in the AREDS and AREDS2 trials (e.g., vitamins C and E, zinc, lutein, and zeaxanthin) demonstrated modest efficacy in reducing the risk of progression to advanced AMD—approximately a 25% reduction in risk on average for intermediate AMD cases [16,69]. However, this benefit is not uniformly observed across all patients, and individual response variability remains a significant issue. Additionally, safety concerns have emerged—for example, β-carotene was excluded from the AREDS2 formulation due to its association with increased lung cancer risk in smokers [16]. A recent review also raised concerns about the limited clinical significance of generalized antioxidant supplementation, highlighting the lack of treatment optimization based on individual biomarkers or genotypes [3].

### 7.2. The Need for Personalized (Precision) Medicine Strategies

Emerging evidence suggests that genetic background significantly influences individual responses to antioxidant therapy. Re-analyses of AREDS data have identified associations between genetic polymorphisms—such as those in *ESRRB-VASH1* or *CFH/ARMS2 loci*—and differential responses to nutritional interventions (Wei et al., 2020) [100]. These findings pave the way for stratified treatment approaches that tailor antioxidant therapy to genetically defined patient subgroups. Thus, precision medicine based on genetic variants and molecular biomarkers is increasingly recognized as a critical strategy for maximizing the therapeutic efficacy of antioxidant interventions in AMD.

### 7.3. Integrative Approaches with Other AMD Therapies

Current AMD treatments, particularly for the neovascular (wet) form, primarily rely on anti-VEGF therapy. However, interventions targeting the underlying pathophysiological mechanisms—oxidative stress, chronic inflammation, and metabolic dysfunction—are equally important. Recent studies have explored combination therapies that simultaneously modulate oxidative, inflammatory, and angiogenic pathways. For example, a combination of triamcinolone acetonide and quercetin has shown efficacy in experimental models by exerting anti-inflammatory, antioxidant, and anti-VEGF effects, thereby reducing intraocular inflammation and choroidal neovascularization [101].

Furthermore, advances in imaging and artificial intelligence (AI)-based analysis offer the possibility of real-time, longitudinal assessment of oxidative stress levels and retinal structural changes. This could enable the development of individualized, multi-phase treatment platforms, integrating nutritional therapy, antioxidants, anti-VEGF agents, and regenerative medicine in a tailored and stepwise manner for each patient.

### 7.4. Strategic Directions for Oxidative Stress Research in AMD

Future research should move beyond generic recommendations and focus on specific, testable priorities. First, there is an urgent need to standardize oxidative stress biomarkers across studies, including lipid peroxidation products (e.g., MDA, 4-HNE), DNA oxidation markers (e.g., 8-OHdG), and antioxidant enzyme activities. Recent work has demonstrated significant inter-cohort variability in oxidative markers, highlighting the need for harmonized methodologies and large-scale validation [102,103].

Second, longitudinal studies are essential to elucidate causal mechanisms. Integrated metabolomic profiling of AMD patients may reveal distinct oxidative stress-related molecular signatures predictive of disease progression [104].

Third, advanced experimental platforms such as patient-derived iPSC-RPE cells offer unprecedented opportunities to dissect oxidative stress responses under genetically controlled conditions [105,106]. These models have already shown promise for drug screening and mechanistic studies in AMD.

Fourth, we propose that precision medicine trials stratified by genetic background (e.g., CFH, ARMS2/HTRA1) be initiated to evaluate differential responses to antioxidant or Nrf2-activating therapies. Such stratification could overcome the heterogeneity observed in prior trials such as AREDS/AREDS2 [9].

Finally, an innovative experimental approach involves real-time imaging of retinal oxidative stress using novel fluorescent probes and adaptive optics technologies. Pilot studies suggest that in vivo detection of ROS and oxidized lipids in human retina is technically feasible and may provide a transformative biomarker for both research and clinical practice [8].

These directions represent actionable priorities that can bridge the gap between basic mechanisms and translational interventions, moving the field toward biomarker-guided, personalized, and mechanism-based therapies for AMD.

### 7.5. Selection of Oxidative Stress Models in In Vitro AMD Studies

Oxidative stress is a central contributor to the pathogenesis of AMD, and its modeling in vitro is crucial for mechanistic studies. Various types of oxidative stress can be applied depending on the research focus. General oxidative stress is commonly induced using hydrogen peroxide or tert-butyl hydroperoxide, which generate ROS and deplete intracellular antioxidants such as GSH, thereby simulating cellular oxidative damage observed in RPE cells [51,107].

Hypoxia-mediated oxidative stress models mimic the low-oxygen environment of the retina, activating hypoxia-inducible factor (HIF) pathways and promoting angiogenic and inflammatory responses relevant to AMD [108]. GSH-dependent models, achieved via inhibition of GSH synthesis, allow examination of the role of endogenous antioxidant defenses in AMD, given that GSH depletion exacerbates ROS-induced cellular injury. Nicotinamide adenine dinucleotide (oxidized form)/nicotinamide adenine dinucleotide (reduced form) (NAD^+^/NADH)-related models focus on the cellular redox state and energy metabolism, as NAD^+^ depletion can impair DNA repair and mitochondrial function, processes implicated in AMD progression [109].

Therefore, the selection of an oxidative stress model should align with the specific mechanistic question being addressed: general oxidative stress for RPE injury and inflammation studies, hypoxia models for angiogenesis and HIF-related pathways, and GSH/NAD^+^-related models to investigate redox homeostasis and mitochondrial resilience. Combining multiple stress paradigms may better reflect the complex oxidative environment in AMD.

### 7.6. Summary and Outlook

Conventional antioxidant therapies have faced challenges due to their limited efficacy, safety concerns, and non-specific application across patient populations. In contrast, a future direction involves the shift toward precision medicine, where genetic polymorphisms and biomarker profiles inform individualized treatment plans. A multimodal approach—anchored in oxidative stress modulation and incorporating anti-VEGF agents, anti-inflammatory drugs, and regenerative strategies—represents a promising next-generation paradigm for AMD treatment. The development of technologies that allow for the visualization and quantification of oxidative stress, combined with molecular profiling, will be pivotal in constructing a multi-layered, integrated therapeutic model. Such a model has the potential to significantly improve AMD prevention, progression control, and long-term preservation of visual function.

## 8. Discussion

This review has comprehensively examined the central role of oxidative stress in the pathogenesis of AMD, from molecular mechanisms to clinical implications. Oxidative stress has been firmly established as a major pathogenic factor contributing to photoreceptor degeneration and retinal structural deterioration through multiple mechanisms, including mitochondrial dysfunction in RPE and photoreceptors, lipid peroxidation, drusen formation, complement system activation, and dysregulation of the Nrf2 pathway [4,19,21,34,37].

### 8.1. Translational Potential of Novel Therapeutic Targets

In addition to current therapies such as AREDS and AREDS2, several promising interventions are emerging from translational research and are being developed toward clinical application. First, mitochondria-targeted antioxidants such as MitoQ and SkQ1 have shown encouraging results in preclinical studies. These agents act directly within the mitochondria to neutralize ROS at their source, potentially mitigating oxidative injury to RPE cells. Their efficacy has been demonstrated in both animal models and in vitro experiments [71,110,111]. Second, Nrf2 activators such as sulforaphane have the potential to reinforce the endogenous antioxidant defense system, thereby interrupting the pathological cycle of oxidative stress, inflammation, and cell death. Neuroprotective effects have been confirmed in several preclinical models [112,113,114]. Third, a multidimensional therapeutic approach is gaining attention. This involves combining oxidative stress modulation with complement inhibition, VEGF blockade, inflammation control, and autophagy regulation. Such integrated strategies may serve as the foundation for future AMD disease-modifying therapies [5,52,115,116].

### 8.2. Prospects for AMD Prevention Through Oxidative Stress Control

Accurate regulation of oxidative stress represents a practical and effective avenue for both preventing AMD onset and slowing disease progression. While the AREDS-based antioxidant supplementation has shown clinical benefit, its limitations—such as the exclusion of β-carotene due to cancer risk, safety concerns, and variability in individual responses—highlight the need for refinement. Future strategies should emphasize precision medicine, incorporating genotype- and biomarker-based patient stratification, and longitudinal monitoring using advanced imaging and proteomic profiling. These tools can enable the development of highly individualized and multi-layered treatment models, tailored to oxidative stress levels and disease stage [5,115].

## 9. Conclusions (Final Remarks)

Oxidative stress is a central pathogenic mechanism in AMD, and deeper understanding of its molecular pathways forms the foundation for innovative therapeutic development. In particular, emerging treatments such as mitochondria-targeted antioxidants and Nrf2 activators hold substantial translational potential, bridging preclinical findings to real-world clinical interventions. The realization of precision and integrative medicine, which combines oxidative stress control with anti-VEGF, anti-inflammatory, and regenerative strategies, represents a realistic and promising pathway toward preventing AMD progression. Looking ahead, a major frontier in AMD research lies in the development of technologies for the visualization and quantification of oxidative stress—via biomarkers and imaging—and the implementation of integrated intervention models. Such advancements will be crucial for sustaining long-term visual function and ultimately preventing blindness in AMD patients.

Oxidative stress is now firmly established as a central driver of the onset and progression of AMD. However, several unresolved issues remain, particularly regarding the standardization of oxidative biomarkers, the long-term efficacy of antioxidant interventions, and the integration of oxidative stress with other pathogenic pathways such as inflammation, complement activation, and lipid metabolism. Importantly, recent evidence suggests that oxidative stress does not act in isolation but interacts dynamically with metabolic and immune processes, underscoring the need for a more integrative framework.

Looking forward, precision medicine approaches that incorporate genetic predispositions (e.g., polymorphisms in oxidative stress-related genes) and biomarker profiles could allow tailored antioxidant strategies for AMD patients. In addition, combining oxidative stress modulation with anti-VEGF therapy, complement inhibition, or regenerative interventions may offer synergistic benefits beyond monotherapy. Finally, novel technologies, including in vivo imaging of oxidative stress and AI-driven risk stratification, hold promise for transforming both diagnosis and therapeutic monitoring. Addressing these challenges will be crucial to translate current mechanistic insights into innovative, clinically effective strategies for AMD management.

## Figures and Tables

**Table 1:** Comparison of Established vs. Emerging Therapies for AMD Targeting Oxidative Stress.

Therapy/Intervention	Mechanism of Action	Clinical Evidence	Development Stage	Key References
**Established Therapies**				
**AREDS/AREDS2 supplementation**	Antioxidants (vitamins C, E, zinc, lutein, zeaxanthin) reduce oxidative burden	Multiple large RCTs (AREDS, AREDS2) demonstrated reduced risk of progression to advanced AMD	Widely recommended in clinical guidelines	Age-Related Eye Disease Study Research Group, 2001; Age-Related Eye Disease Study 2 Research Group, 2013 [16,69]
**Anti-VEGF therapy**	Blocks VEGF-mediated neovascularization, indirectly reducing oxidative stress from hypoxia	Numerous RCTs show efficacy in wet AMD	Standard of care	Rosenfeld et al., 2006; Heier et al., 2012 [94,95]
**Emerging Therapies**				
**Saffron (crocin, crocetin)**	Antioxidant, neuroprotective, mitochondrial protection	Several small-to-moderate RCTs show improvements in visual function	Phase II/III clinical evaluation ongoing	Maccarone et al., 2008; Broadhead et al., 2019; Heydari et al., 2023 [30,79,80]
**L-DOPA repositioning**	Enhances melanin synthesis in RPE, antioxidant and cytoprotective effects	Epidemiological evidence (reduced AMD incidence in Parkinson’s patients on L-DOPA)	Repurposing under investigation	Brilliant et al., 2016 [81]
**Cerium oxide nanoparticles (CeO_2_-NPs)**	Regenerative catalytic antioxidant, anti-inflammatory, prevents drusen formation	Strong preclinical evidence in animal models	Preclinical, early translational	Fiorani et al., 2015; Maccarone et al., 2020 [88,89]
**Mitochondria-targeted antioxidants (e.g., MitoQ, SkQ1)**	Scavenge ROS directly in mitochondria	Preclinical and early-phase trials report efficacy	Early-phase human trials	Skulachev et al., 2012; Gioscia-Ryan et al., 2014 [96,97]
**Nrf2 activators (e.g., sulforaphane)**	Enhance endogenous antioxidant defense pathways	Preclinical neuroprotective evidence	Early-phase translational studies	Gao et al., 2004; Sachdeva et al., 2014 [98,99]

## Data Availability

No new data were created or analyzed in this study. Data sharing is not applicable to this article.

## Abbreviations

The following abbreviations are used in this manuscript:

4-HNE	4-hydroxy-2-nonenal
8-OHdG	8-hydroxy-2′-deoxyguanosine
A2E	N-retinylidene-N-retinylethanolamine
AGE	Advanced glycation end product
AI	Artificial intelligence
ALE	Advanced lipoxidation end product
AMD	Age-related macular degeneration
APOA1	Apolipoprotein A1
APOE	Apolipoprotein E
AREDS	Age-Related Eye Disease Study
ARMS2	Age-related maculopathy susceptibility 2
CDDO-Me	the methyl ester of 2-cyano-3,12-dioxooleana-1,9(11)-dien-28-oic acid
CeO2-NPs	Cerium oxide nanoparticles
CFB	Complement factor B
CFH	Complement factor H
DAMP	Danger-associated molecular pattern
DHA	Docosahexaenoic acid
DNA	Deoxyribonucleic acid
EPA	Eicosapentaenoic acid
GPX4	Glutathione peroxidase 4
GSH	γ-glutamyl-cysteinyl-glycine (glutathione) GSH)
GST	Glutathione S-transferase
HDL	High-density lipoprotein
HIF	Hypoxia-inducible factor
HO-1	Heme oxygenase-1
HTRA1	HtrA serine peptidase 1
IL	Interleukin
iPSC	Induced pluripotent stem cell
LCN1	Lipocalin-1
L-DOPA	Levodopa
LIPC	Hepatic Lipase (gene)
MDA	Malondialdehyde
MitoQ	Mitoquinone
mtDNA	Mitochondrial DNA
mTOR	Mammalian target of rapamycin
NAD^+^	Nicotinamide adenine dinucleotide (oxidized form)
NADH	Nicotinamide adenine dinucleotide (reduced form)
NFE2L2	Gene name of nuclear factor erythroid 2-related factor 2 (Nrf2)
NF-κB	Nuclear factor kappa B
NO_2_	Nitrogen dioxide
Nrf2	Nuclear factor erythroid 2-related factor 2
O_3_	Ozon
OCT	Optical coherence tomography
oxLDL	Oxidized low-density lipoprotein
OXYS rats	Oxidative stress-prone rats
PM_2.5_	Particulate Matter with an aerodynamic diameter of less than 2.5 μm
PGC-1α	Peroxisome proliferator–activated receptor gamma coactivator-1 alpha
PUFAs	Polyunsaturated fatty acids
RAGE	Receptor for advanced glycation end products
RNA	Ribonucleic acid
ROS	Reactive oxygen species
RPE	Retinal pigment epithelium
SkQ1	Skulachev Ion-1
SMOC2	Secreted protein, acidic and rich in cysteine (SPARC) related modular calcium binding 2
SOD	Superoxide dismutase
SPARC	Secreted protein, acidic and rich in cysteine
SUCNR1	Succinate receptor 1
TAS	Total antioxidant status
TF	Serotransferrin
TPP^+^	Triphenylphosphonium
UV	Ultraviolet
VEGF	Vascular endothelial growth factor
VOC	Volatile organic compound
